# Urinary Sex Steroids and Anthropometric Markers of Puberty - A Novel Approach to Characterising Within-Person Changes of Puberty Hormones

**DOI:** 10.1371/journal.pone.0143555

**Published:** 2015-11-23

**Authors:** Gurmeet K. S. Singh, Ben W. R. Balzer, Patrick J. Kelly, Karen Paxton, Catherine I. Hawke, David J. Handelsman, Katharine S. Steinbeck

**Affiliations:** 1 ANZAC Research Institute, University of Sydney, Camperdown, NSW, Australia; 2 Faculty of Pharmacy, Universiti Teknologi MARA, Selangor, Malaysia; 3 Academic Department of Adolescent Medicine, Children’s Hospital at Westmead, Westmead, NSW, Australia; 4 Discipline of Paediatrics and Child Health, Sydney Medical School, University of Sydney, Camperdown, NSW, Australia; 5 Sydney School of Public Health, Sydney Medical School, University of Sydney, Camperdown, NSW, Australia; 6 School of Rural Health, Sydney Medical School, University of Sydney, Camperdown, NSW, Australia; John Hopkins University School of Medicine, UNITED STATES

## Abstract

**Background/Aims:**

The longitudinal relationships of within-individual hormone and anthropometric changes during puberty have not ever been fully described. The objectives of this study were to demonstrate that 3 monthly urine collection was feasible in young adolescents and to utilise liquid chromatography-tandem mass spectrometry assay methods for serum and urine testosterone (T), estradiol (E_2_) and luteinizing hormone (LH) in adolescents by relating temporal changes in urine and serum hormones over 12 months to standard measures of pubertal development.

**Methods:**

A community sample of 104 adolescents (57 female) was studied over 12 months with annual anthropometric assessment, blood sampling and self-rated Tanner staging and urine collected every 3 months. Serum and urine sex steroids (T, E_2_) were measured by liquid chromatography-tandem mass spectrometry (LC-MS/MS) and LH by immunoassay.

**Results:**

A high proportion (92%) of scheduled samples were obtained with low attrition rate of 6.7% over the 12 months. Urine hormone measurements correlated cross-sectionally and longitudinally with age, anthropometry and Tanner stage.

**Conclusion:**

We have developed a feasible and valid sampling methodology and measurements for puberty hormones in urine, which allows a sampling frequency by which individual pubertal progression in adolescents can be described in depth.

## Introduction

The circulating gonadotropin, testosterone (T), and estradiol (E_2_) changes that drive the external manifestations of puberty are well described from cross-sectional studies according to chronological age or Tanner staging interpreted quasi-longitudinally [1, 2]. Such cross-sectional analysis artificially smooths longitudinal data due to a low resolution in temporal sampling, markedly underestimating the underlying within-subject variability. Combined with this variation in individual hormones is the normal variability in both time of onset and tempo of completing puberty. Both timing and tempo of hormone change might be an important intermediate factor in the marked behavioural and psychological changes of adolescence, but presents challenges in its study. Previous studies that have considered how the individual variability in puberty hormone change might influence the dramatic psycho-bio-behavioural changes in adolescence relied upon older methods of often direct (unextracted) sex steroid immunoassays. This is a less accurate technology especially at low circulating steroid levels concentrations [3–5], and is now being supplanted by more sensitive and specific liquid chromatography-tandem mass spectrometry (LC-MS/MS)-based steroid assays [3]. Similar more sensitive LC-MS/MS -based methods have been recently described for serum sex steroids in pre-pubertal children [6].

In clinical settings the usual methods to appraise pubertal development and, by implication, its variability, comprise hormone measurements and anthropometry, with emphasis on timing of the height growth spurt, clinical inspection and rating of secondary sexual characteristics and bone age [7, 8]. In epidemiological studies and community studies the definition of puberty has to be simplified to be based on adolescent self-report or parental report against either Tanner stage line drawings [9, 10] or Petersen’s Pubertal Development Scale [1]. Self-rated Tanner staging is less intrusive and often the only Ethics Board approved method to assess puberty as defined by secondary sexual characteristics for epidemiological and community studies involving adolescents who remain legally minors. This has to sacrifice some reliability, especially in early puberty, and with the limited number of developmental stages available to select [11–14]. Recent work has demonstrated self-rating is of insufficient accuracy to be of use in the clinical setting, however may be acceptable in research settings where clinician assessment is not possible [15]. Menarche (a late pubertal event), spermarche [16, 17] which is difficult to evaluate [18], and voice breakage have all been described in relation to age and/or Tanner stage [19, 20]. However, as singular time points these provide minimal information on either timing of onset or tempo of puberty. To date in the literature there have been no reports describing individual puberty hormone change with a sufficient measurement frequency to adequately capture individual variation. Unless this is achieved, it is not possible to determine the true effects of puberty hormones on adolescent mood, wellbeing and behaviour.

This study aims to examine the feasibility of frequent three-monthly urine collection in a large Australian adolescent cohort, as well as the utility of LC-MS/MS assays urine and serum sex hormones. Additionally, we will describe changes in E_2,_ T (using these LC-MS/MS) assays and luteinizing hormone (LH) over 12 months in adolescents to compare results for urine and serum hormones. Finally, we aim to examine the association between the changes in anthropometry and self-reported Tanner stage with changes in hormones in both urine and serum.

## Materials and Methods

### Setting and Participants

The study was set in two regional towns in the state of New South Wales (NSW), Australia. Adolescents between the ages of 10 and 12 years were recruited from local schools. Fasting morning blood samples were collected at 0 and 12 months for the measurement of E_2_, T and LH and first morning (fasting) urine collected three monthly for the same measures. No participants had an endocrine disorder or were on any type of gonadal steroid hormone therapy.

### Anthropometry and pubertal staging

Height was measured using a portable stadiometer (to 0.1 cm). Weight was measured in light clothing using a Tanita TBF-300 Pro Body Composition Analyzer [21]. Body mass index (BMI) was calculated using these measures. The adolescents provided a self-rating of puberty using line drawings based on the Tanner stages [9, 10]. Females provided self-rating of breast stage and males provided self-rating of genitalia stage. Self-report of Tanner stage [11–14] was the only feasible and ethically acceptable measure of pubertal staging available to the investigators. This situation is now a common limitation, as both Institutional Review Boards and parents are reluctant to permit direct physical examination of undressed healthy children for a research study of normal puberty.

### Hormone measurements

Following a 12 hour fast urine was collected at home before blood samples were collected between 7:00 am and 8:30 am in order to minimise the effects of diurnal hormone variation [1, 22]. The urine sample was collected as a first morning void into a lidded container and immediately placed into an insulated carry bag on an ice brick. This was refrigerated in a freezer at -20 C within 3 hours. Blood and urine samples were then transported on dry ice to the research laboratory where these were kept at -80 C until analysed–approximately 12 months later. Post-menarcheal girls provided urine and blood specimens in the mid-follicular phase (Day 7–10) of their menstrual cycle in order to standardise collection time. Urine and serum steroids were measured by liquid chromatography, tandem mass spectrometry (LC-MS/MS) as modified from a previously described method for serum [23] and adapted for urine specimens following enzymatic deconjugation. The conjugated steroid underwent hydrolysis using β-glucuronidase enzyme from *Escherichia coli* K12 (Roche Diagnostic, Mannheim, Germany) that deconjugates the glucuronides moiety from steroids. The developed LC-MS/MS method measures unconjugated steroids (i.e. originally unconjugated plus deconjugated). The details of the novel urine assays have been published [24]. Briefly, urine and serum specimens were separated by liquid chromatrography using a Shimadzu Nexera UHPLC system (Shmadzu Scientific Instruments, Columbia, MD). Following this, tandem-mass spectrometry analysis was performed on samples using an API-5000 triple-quadrupole mass spectrometer (Applied Biosystem/MDS SCIEX, Ontario, Canada) [24]. A thorough validation was carried out for the LC-MS/MS method to measure E_2_ and T according to standard FDA/EMEA analytical validity criteria.

The calibration curves of the urinary steroid LC-MS/MS method ranged from 0.025 and 32 ng/mL for T and 0.05 and 32 ng/mL for E_2_, and fitted quadratic functions with r of 0.999 or better. Within-day and between-day accuracies and precision at all levels of quality control ranged from 95–105% and 2.6–9.7%, respectively. Using a 500 μL urine sample the limits of detection (LOD) and limits of quantitation (LOQ), respectively, were 5 pg/mL and 25 pg/mL for T and 25 pg/mL and 50 pg/mL for E_2_. Matrix effects were negligible with no significant ion suppression or enhancement for either analyte with recovery values between 102–108%. Extraction recovery and process efficiency were between 93–103% for both the analytes at all levels of QC. The β-glucuronidase enzyme hydrolysis (deconjugation) efficiency was 100–102% after an overnight incubation at room temperature.

Specificity of both the analytes was tested against structurally related compounds that potentially may interfere with the method, including estrone, epitestosterone, androsterone, etiocholanolone, epietiocholanolone, 3-α androstanediol, 3-β androstanediol, and dehydroepiandrosterone. All the steroids listed did not interfere with the studied analytes. Blood and urine samples with values less than the lower limit of quantitation (LLOQ) for E_2_ and T were taken as half the LLOQ. Total of 27 urine (E_2_: 16, T: 11) and 2 serum (T) samples were below the LLOQ. Serum and urine LH were measured by Immulite 1000 LH (Siemens) which detects intact LH and LHβ subunit and provided reproducible measurements in frozen stored urine[25]. The within-assay coefficients of variation were <10%. Serum and urine LH values below the detection limit (0.1 IU/L) were set at zero (10 serum and 5 urine samples). Urine FSH assays (Immulite, Delfia) did not pass validity tests (dilutional linearity, quantitative spike recovery) and were therefore not used in this study. All urine hormone concentrations were adjusted for urine specific gravity (SG) measured by reagent strip (ChoiceLine 10, Roche Diagnostics) to a standard SG of 1.020.

### Statistical analyses

Anthropometric measurements and hormones levels (blood and urine) were summarised by gender over time using means and standard deviations for continuous variables and frequencies and proportions for categorical variables. Linear mixed effects models were used to assess longitudinal changes in hormones, with gender and collection time as covariates. A random effect for child was included in these models. Interaction between gender and collection time was tested for each hormone. Linear regression was used to analyse the association between changes in anthropometry (height, weight, BMI) and self-rated Tanner stage and changes in urine and serum hormones. For these, the models were adjusted for the baseline anthropometric measurement and baseline hormone concentration. All p-values were calculated using Wald tests. Statistical analyses were conducted using Stata 12.1 (StataCorp, Texas, USA). Statistical significance was set at 0.05.

### Ethical considerations

The study has ethical approval from the Human Research Ethics Committee, University of Sydney (HREC 13094) within the National Health and Medical Research Council Guidelines for Human Experimentation, which is consistent with the Declaration of Helsinki. All participants assented, and a parent provided written informed consent prior to commencing the study.

## Results

### Cohort Characteristics

One hundred and four participants were recruited. The mean ages (SD) for the study participants at baseline were 12.5 (0.93) years for males and 11.8 (0.98) years for females. At follow-up, the ages were 13.5 (0.94) years for males and 12.9 (0.97) years for females. Anthropometric characteristics for this cohort are shown in Table 1. For the females, 22 (39%) had menarche prior to the study and one additional girl experienced menarche during the follow-up year. Post-menarcheal girls were significantly older than their pre-menarcheal counterparts (12.9 years vs. 11.9 years, p<0.001). A high proportion of scheduled samples were collected for urine (484, 92%) and serum (194, 93%). There was a low loss to follow-up (7, 6.7%). For those not lost to follow-up, 16 females and 12 males did not provide at least one urine specimen. There was no difference in age between those who provided a specimen and those who did not (mean difference 0.35 years; 95% CI -0.21–0.92; p = 0.23), nor was there a difference in self-rated Tanner stage (p = 0.53).

**Table 1:** Baseline and 12-month follow-up anthropometry measurements.

	Baseline		12 Months		p-value		
Mean (SD)	Male (n = 47)	Female (n = 57)	Male (n = 47)	Female (n = 57)	Gender*	Time*	Gender x Time
Height (cm)	156.8 (9.9)	150.8 (8.5)	163.8 (10.1)	157.1 (7.9)	**<0.001**	**<0.001**	0.18
Weight (kg)	49.3 (12.0)	44.1 (10.9)	55.2 (13.2)	50.1 (11.4)	**0.026**	**<0.001**	0.98
BMI (kg/m^2^)	19.7 (3.5)	19.3 (3.7)	20.4 (3.8)	20.3 (4.0)	0.56	**<0.001**	0.16
Self-rated Tanner stage	3 (1.2)	2 (1.1)	4 (1.0)	3 (1.1)	**0.001**	**<0.001**	0.72

* these p-values were calculated with both gender and time in the model, but with no interaction term

Mean anthropometric measurements (Table 1) increased significantly over 12 months whereas age- and gender-standardized z-scores did not change over the 12 months follow-up. Pre-menarcheal girls significantly increased their age-standardized weight (p<0.001) and height z-scores (p = 0.002), whereas these z-scores did not change for post-menarcheal girls. Rate of change in anthropometric measurements were similar between genders (interaction p>0.05).

Self-rated Tanner staging increased with fewer in stage 1 and more in stage 5 at 12-month follow-up (Table 2). During the year, 14 participants (13.5%) progressed two Tanner stages, 43 (41.3%) progressed one stage and 41 (39.4%) did not change in their self-rated Tanner stage. Six (5.8%) participants (three boys) provided a lower self-rated Tanner stage at follow-up than baseline. One participant (1%, 1 boy) did not provide baseline Tanner staging and seven (6.7%, 1 boy, 6 girls) did not provide follow-up Tanner staging.

**Table 2:** Self-Rated Tanner staging at baseline and 12-month follow-up.

N	Baseline		12 Months	
Tanner	Male (%)	Female (%)	Male (%)	Female (%)
1	6 (13)	14 (25)	0 (0)	3 (6)
2	8 (17)	17 (30)	7 (16)	13 (25)
3	9 (20)	16 (28)	6 (13)	15 (29)
4	18 (39)	7 (12)	18 (40)	12 (24)
5	5 (11)	3 (5)	14 (31)	8 (16)
Total	46	57	45	51

### Hormone measurements

#### Urine hormone levels versus time

For the 484 urine collections, 14 (3%) E_2_, nine (2%) T and five (1%) LH assays were below the LLOQ. For LH, these participants were younger than the rest of the cohort (mean difference 1.35 years; 95% CI 0.41–2.30; p = 0.005), but there was no age difference between those with urine E_2_ or T samples below or above the limits of detection. In five such cases (one urine E_2_, one urine E_2_ and T, one urine T, one urine and serum and one urine LH) the participant provided a Tanner stage 1 at baseline.

**Fig 1:**
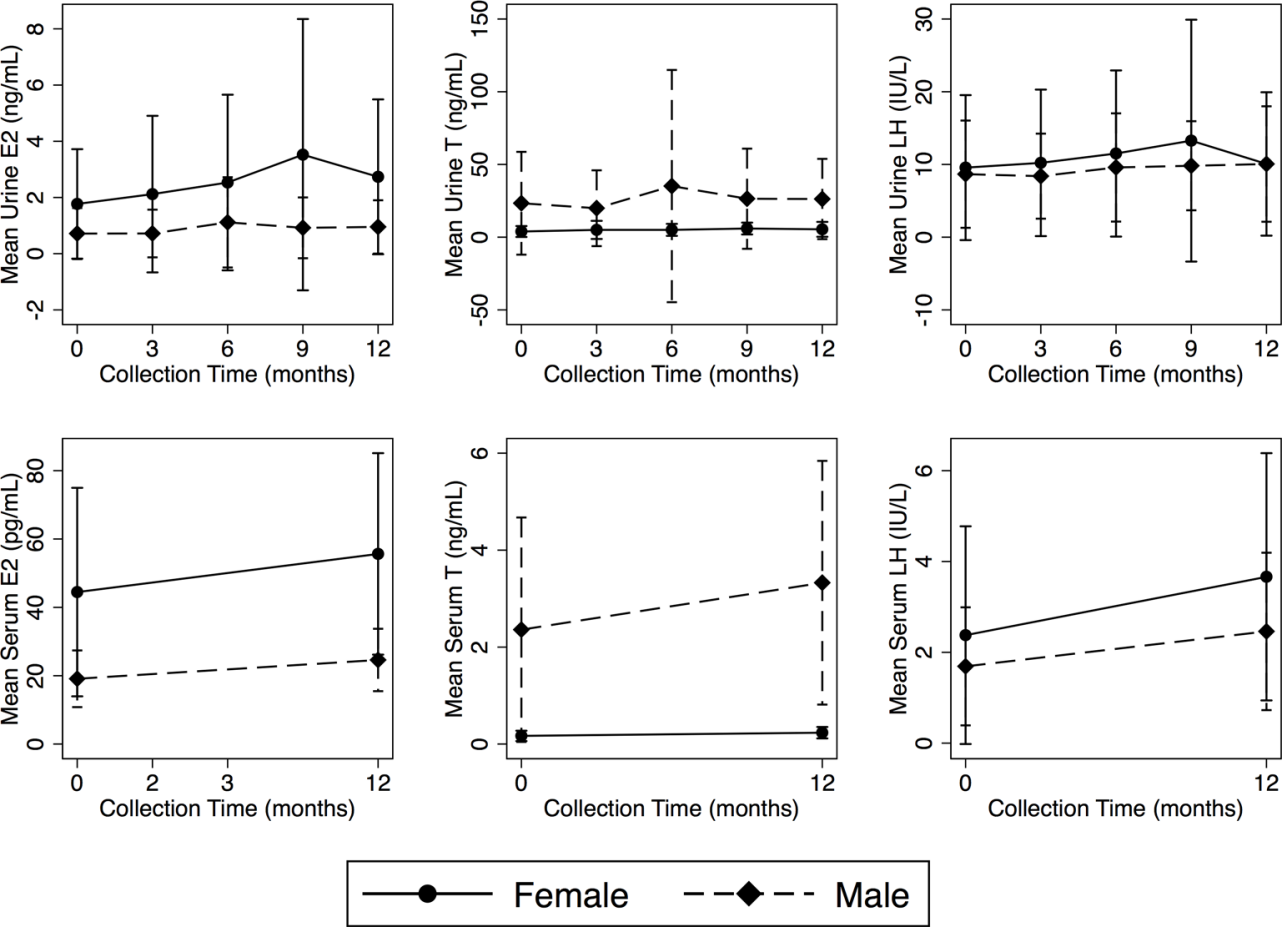
Plots of mean and SD of urinary E_2_, T and LH concentrations derived from 3, 6, 9 and 12 months collection changes from baseline (0) for urine (top) and serum (bottom).

Fig 1 shows within-person changes from baseline over the 12-month collection period and Table 3 shows the mean three monthly values for urinary E_2_, T and LH over 12 months. There is a significant increase and clear upward pattern for all subjects in mean urine T and E_2_ plotted over time from baseline to follow-up in E_2_, T and LH. The same pattern is seen in females when stratified by menarcheal status (data not shown). Urine hormone levels were not strictly progressive and in some instances decreased over time, though there was an overall increase in mean levels for all three hormones for males and females. For example, there was a 22.2% decrease in mean male and a 40.0% decrease in mean female E_2_ between six and nine months, a 14.6% decrease in male T between baseline and three months, a 24.6% decrease for male and 16.7% decrease for female T between six and nine months. For LH, there was a decrease for males between baseline and three months of 3.4% and between six and nine months a 14.3% decrease and a 23.3% decrease for males and females, respectively. At all other times there was an increase in urine hormones. For serum hormones, a decline from baseline to follow-up was observed 22 adolescents (11 female) for E_2_, 19 (13 female) for T and in 26 adolescents (18 female) for LH. Overall declines were observed in 23 (9 female) for urinary E_2_, 24 (14 female) for urinary T and in 48 adolescents (30 female) for urinary LH.

**Table 3:** Mean urine E_2_, T and LH over time for males (M) and females (F).

Mean (SD)	Baseline		3 Months		6 Months		9 Months		12 Months (Follow-Up)		p-value		
	M	F	M	F	M	F	M	F	M	F	Gender*	Time*	Gender x Time
E_2_ (ng/mL)	0.7 (0.9)	1.8 (2.0)	0.7 (0.9)	2.1 (2.8)	1.1 (1.6)	2.5 (3.1)	0.9 (1.1)	3.5 (4.8)	1.0 (1.0)	2.7 (2.8)	**0.002**	**0.002**	0.18
T (ng/mL)	23.3 (35)	3.9 (3.8)	19.9 (26.1)	5.0 (6.2)	35.1 (80)	5.0 (4.1)	26.4 (34)	6.0 (4.1)	26.2 (28)	5.4 (5.1)	**0.024**	**<0.001**	**0.03**
LH (IU/L)	8.7 (7.4)	9.6 (10.0)	8.4 (5.9)	10.2 (10.1)	9.6 (7.4)	11.5 (11.4)	9.8 (6.1)	13.3 (16.6)	10.1 (7.9)	10.1 (9.9)	0.232	**0.001**	0.82

* these p-values were calculated with both gender and time in the model, but with no interaction term

#### Urine versus serum

Urinary E_2_, T and LH all correlated with their serum hormone measurements (p<0.001 for all; see Table 4), and increased in each Tanner stage.

**Table 4:** Pearson’s Correlation Coefficients for Serum and Urinary Hormones using 0 and 12 month data.

	Urine
**Baseline**	
Serum E2	0.7089
Serum T	–0.7864
Serum LH	–0.5513
**Follow-Up**	
Serum E2	0.7245
Serum T	0.7918
Serum LH	–0.4206
**Pooled**	
Serum E2	0.7187
Serum T	0.7746
Serum LH	–0.4735

p<0.001 for all

#### Serum hormone levels versus time

Mean serum hormone levels at baseline and one year follow-up are shown in Table 5. Of the 194 serum collections, two (1%) T and nine (5%) LH assays (all in separate individuals) were below the LLOQ. No E_2_ samples were below LLOQ. Those with LH samples below the limits of detection were significantly younger than their peers (mean difference = 1.47 years 95% CI 0.81–2.14 p<0.001). No difference in age was observed for those with serum E_2_ or T samples below limits of detection.

**Table 5:** Baseline and 12-month follow-up serum E_2_, T and LH.

Mean (SD)	Baseline		12 months		p-value		
	Male (n = 47)	Female (n = 57)	Male (n = 47)	Female (n = 57)	Gender*	Time*	Gender x Time
E_2_ (pg/mL)	19.1 (8.5)	43.4 (29)	24.9 (9)	54.7 (30)	**<0.001**	**<0.001**	0.17
T (ng/mL)	2.4 (2.3)	0.16 (0.10)	3.4 (2.55)	0.2 (0.12)	**<0.001**	**<0.001**	**0.03**
LH (IU/L)	1.7 (1.3)	2.3 (2.4)	2.5 (1.8)	3.6 (2.7)	0.23	**0.001**	0.82

* these p-values were calculated with both gender and time in the model, but with no interaction term

Hormone values all significantly increased over the 12-month period and were significantly different between genders, but rate of change was not statistically significant between genders (interaction p>0.05), except for T.

### Association between Anthropometry Measurements and Urine and Serum Hormones

Urinary E_2_, T and LH all positively correlated with Tanner staging at baseline and 12-month follow-up (p<0.001 for all). Table 6 shows the associations between anthropometry changes and changes in sex hormones. Change in height was associated with changes in serum T, and serum and urine LH in females and both serum and urine T in males. Change in weight was associated with changes in urine E_2_, serum T and serum LH in females. Serum T and LH was associated with self-rated Tanner stage in males, but this was not statistically significant based on the urine samples. No other significant associations were observed between serum or urinary hormones and changes in anthropometry over 12 months.

**Table 6:** Regression results for serum (left) and urine (right) hormones and anthropometric markers of puberty.

Serum	R^2^	β	p-value	Urine	R^2^	β	p-value
**Change in Height**							
**Female**							
E_2_	0.15	-0.001	0.94	E_2_	0.25	0.26	0.24
T	0.22	10.19	**0.035**	T	0.22	0.13	0.24
LH	0.32	0.52	**0.001**	LH	0.24	0.09	**0.045**
**Male**							
E_2_	0.15	0.09	0.12	E_2_	0.16	0.61	0.22
T	0.34	0.89	**<0.001**	T	0.15	0.04	**0.019**
LH	0.02	-0.16	0.68	LH	0.08	0.04	0.44
**Change in Weight**							
**Female**							
E_2_	0.03	-0.01	0.66	E_2_	0.15	0.55	**0.034**
T	0.08	11.81	**0.031**	T	0.12	0.21	0.10
LH	0.26	0.45	**0.013**	LH	0.07	0.08	0.14
**Male**							
E_2_	0.03	0.07	0.58	E_2_	0.06	1.56	0.16
T	0.04	0.21	0.69	T	0.06	0.05	0.13
LH	0.01	-0.10	0.90	LH	0.003	-0.01	0.88
**Change in BMI**							
**Female**							
E_2_	0.04	0.001	0.92	E_2_	0.07	0.12	0.20
T	0.07	2.18	0.27	T	0.08	0.03	0.48
LH	0.16	0.03	0.63	LH	0.03	0.01	0.69
**Male**							
E_2_	0.04	-0.02	0.41	E_2_	0.02	0.05	0.82
T	0.46	-0.01	0.96	T	0.01	0.003	0.63
LH	0.02	-0.10	0.58	LH	0.22	0.02	0.11
**Change in Self-Rated Tanner Stage**							
**Female**							
E_2_	0.19	0.005	0.40	E_2_	0.23	0.10	0.11
T	0.23	2.18	0.15	T	0.19	0.03	0.31
LH	0.26	0.10	0.06	LH	0.20	0.02	0.36
**Male**							
E_2_	0.39	0.04	**0.042**	E_2_	0.31	0.20	0.22
T	0.46	0.18	**0.008**	T	0.35	0.01	0.13
LH	0.29	-0.06	0.62	LH	0.36	0.02	0.21

**Fig 2:**
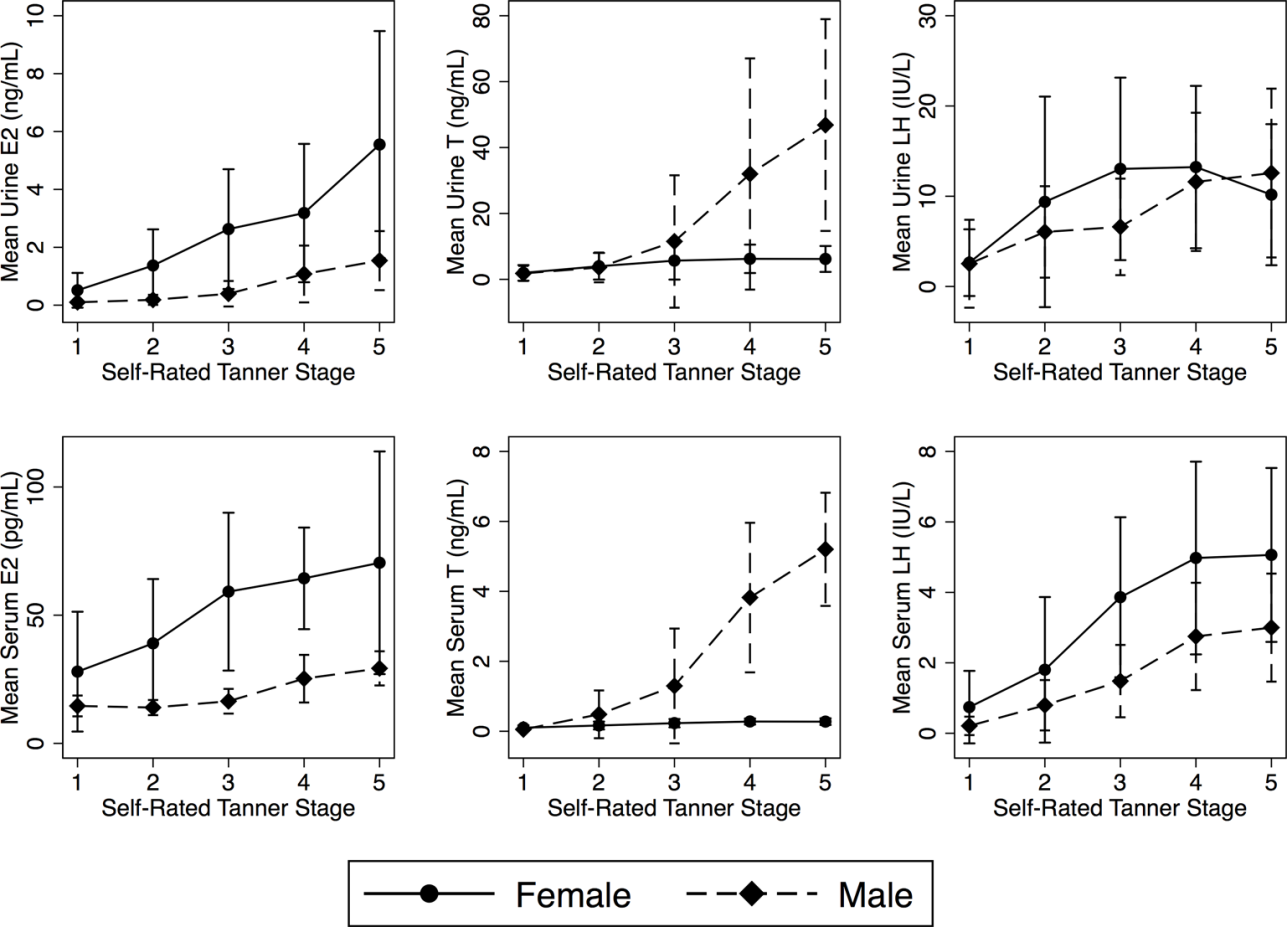
Plots of mean and SD E_2_ (left), T (centre) and LH (right) in urine (top) and serum (bottom) specimens, by Tanner stage.

Fig 2 shows urine and serum E_2_, T and LH stratified by Tanner stages. Data stratified by chronological age were similar. Hormone concentrations increased through each Tanner stage and each year of age.

## Discussion

There are two major findings of this study. First is the demonstration that it is feasible to collect urine samples from a community-based adolescent cohort at three-monthly intervals, with high compliance (93% serum and 92% urine collections completed) and low follow-up attrition rate. Second is the finding that urine levels both correlate with serum testosterone and oestradiol and have the potential to provide a more subtle and nuanced description of individual puberty hormone progression.

In order to demonstrate the specific effects of puberty hormones on any biological aspect of adolescent development and health, a methodology that allows more frequent biological sampling than has ever been previously reported for community based field studies is essential. A single reading of testosterone or oestradiol in whatever biological sample is of limited use. It is change that is relevant and hence the importance of longitudinal samples with frequent sampling. Urine samples have the advantage over blood samples that these are more acceptable to both research ethics and adolescents. Each overnight sample also provides a more time-integrated hormone measure. This is particularly true in early puberty when pubertal hormones commence pulsatile secretion nocturnally [1], so that a morning overnight urine sample may be more informative than a serum sample at any single time point. Urine collections also allow for more frequent collection than repeated venepuncture would be tolerated [26]. Salivary samples are potentially easier to collect, but blood contamination and influence of flow-rate on measurements seriously limits validity and accuracy and these may be subject to non-compliance by children [27].

Urine steroid measurements have been dramatically improved upon by introduction of new LC-MS/MS technology. Urine steroid LC-MS/MS measurements have been used exclusively as the basis of for anti-doping tests worldwide for the last few decades [3] and were required to have high level specificity. As a result this led to the recognition of the lower specificity of steroid immunoassays and resulted in major changes to clinical endocrinology research [3]. Steroid mass spectrometry ensures the accuracy and specificity necessary for the measurement of low levels of E_2_ and T and the detection of subtle changes in these gonadal steroids over time.

Anthropometric and serum hormone changes in our study revealed the anticipated increases over the course of one year in a cohort of young adolescents. The urine data also revealed anticipated hormone increases over the 12-months of observation. However, mean and individual urinary hormone changes were not strictly progressive, suggesting within-subject variability in early and mid-pubertal hormone levels, which may contain novel and hitherto unexploited information on determinants of biological aspects of pubertal progression. As these urine data are novel we are unable to compare with any previously published data, notably the degree of individual variability. Mouritsen and colleagues have shown that serum testosterone is variable in a longitudinal study of 20 adolescents (10 male) with sample collection biannually for five years [28]. Individual hormone curves showed testosterone levels fluctuated during the study, though the overall pattern demonstrated an increase in serum hormones testosterone (as assayed by LC-MS/MS and immunoassay) from recruitment to the end of follow-up. Biro’s work in 252 females followed every six months for six years similarly demonstrated androstenedione, estrone, E_2_ and T by LC-MS, with changes which increased over the transition from pre-puberty to six months after reaching Tanner stage 2 [29]; however, neither provide inter-individual variability or mean inter-individual hormone levels. Both previous studies measured only serum steroids in a single gender. We also identify the need for studies of longer duration in order to comment on what implications this variability (or what could be interpreted as instability) of hormone change may have on more distal responses to gonadal hormone change patterns in puberty be these physical, neurobehavioral or mood. This finding also suggests that three monthly urine collections over an extended period have the capacity to provide new insights into the biology of puberty.

We have shown considerable overlap between hormone levels at each age and self-reported Tanner stage, which emphasises the need for better descriptors of *individual* puberty hormone changes. Such overlap is consistent with other data using LC-MS/MS and clinician rating [30–32] and indicate the complexity and dynamic nature of puberty. Both Tanner stage and anthropometric change lag behind hormonal change. Using the former as surrogates for puberty hormone change will less accurately describe the relationship between hormone changes and the resultant physical changes, as well as any other adolescent health or developmental change of interest, such as mood or behaviour. More broadly still, this study has potential importance to the understanding of normal puberty because there is a paucity of frequently sampled longitudinal studies of the hormonal changes during puberty. This situation leads to over-interpretation of how adolescent mood, behaviour and wellbeing relate to puberty hormones, going well beyond available data [33, 34]. Our study methodology with frequent sampling of an easily accessible biological fluid is likely provide new insights into the dynamics of pubertal hormonal changes, especially in the tempo and stability of puberty change. These data in turn may allow an understanding of how puberty hormone change relates to the behavioural aspects of adolescence.

Previous work has questioned the validity of self-rated Tanner staging [1, 11–14]. In this study a high proportion of adolescents completed self-assessment with findings of stable or an advance in Tanner stage at 12 months follow-up in all but 6% of adolescents (who went backwards in a Stage) Self-rated Tanner staging also corresponded well with conventional anthropometric measures of puberty change.

In conclusion, our work has established a feasible method for intensive urine sampling of community-dwelling adolescents and used a robust methodology of urine sex steroid hormone measurement, using liquid chromatography-tandem mass spectrometry measurements for urine sex steroids [3], which display the high sensitivity and specificity to detect lower levels of sex hormones, a particular challenge to the study of pubertal progression [6, 35]. Based on previously recorded longitudinal growth data [36, 37], it is anticipated that frequently measured urine samples over the two to three year window of normal puberty might well provide a firmer biological basis for clinically observed patterns of puberty, such as rapid, slow or variable tempo, which may over the longer term support some of the observed differences in adolescent mood and behaviour.

## Supporting Information

S1 Dataset (XLSX)

## References

[1] Dorn LD, Dahl RE, Woodward HR, Biro F. Defining the boundaries of early adolescence: A user's guide to assessing pubertal status and pubertal timing in research with adolescents. Applied Developmental Science. 2006;10(1):30–56. 10.1207/s1532480xads1001_3

[2] Lee PA, Houk CP. Puberty and Its Disorders In: Lifschitz F, editor. Pediatric Endocrinology. New York: Informa Healthcare; 2006 p. 273–303.

[3] Handelsman DJ, Wartofsky L. Requirement for mass spectrometry sex steroid assays in the journal of clinical endocrinology and metabolism. The Journal of clinical endocrinology and metabolism. 2013;98(10):3971–3. 10.1210/jc.2013-3375 . 24098015

[4] Sikaris K, McLachlan RI, Kazlauskas R, de Kretser D, Holden CA, Handelsman DJ. Reproductive hormone reference intervals for healthy fertile young men: evaluation of automated platform assays. The Journal of clinical endocrinology and metabolism. 2005;90(11):5928–36. 10.1210/jc.2005-0962 . 16118337

[5] Taieb J, Mathian B, Millot F, Patricot MC, Mathieu E, Queyrel N, et al Testosterone measured by 10 immunoassays and by isotope-dilution gas chromatography-mass spectrometry in sera from 116 men, women, and children. Clin Chem. 2003;49(8):1381–95. . 12881456 10.1373/49.8.1381

[6] Courant F, Aksglaede L, Antignac J-P, Monteau F, Sorensen K, Andersson A-M, et al Assessment of Circulating Sex Steroid Levels in Prepubertal and Pubertal Boys and Girls by a Novel Ultrasensitive Gas Chromatography-Tandem Mass Spectrometry Method. Journal of Clinical Endocrinology & Metabolism. 2010;95(1):82–92. 10.1210/jc.2009-1140 19933393

[7] Biro FM, Lucky AW, Huster GA, Morrison JA. Pubertal staging in boys. J Pediatr. 1995;127(1):100–2. . 7608791 10.1016/s0022-3476(95)70265-2

[8] Bordini B, Rosenfield RL. Normal pubertal development: part II: clinical aspects of puberty. Pediatrics in review / American Academy of Pediatrics. 2011;32(7):281–92. Epub 2011/07/05. 10.1542/pir.32-7-281 . 21724902

[9] Marshall WA, Tanner JM. Variations in pattern of pubertal changes in girls. Arch Dis Child. 1969;44(235):291–303. Epub 1969/06/01. 5785179 10.1136/adc.44.235.291 PMC2020314

[10] Marshall WA, Tanner JM. Variations in the pattern of pubertal changes in boys. Arch Dis Child. 1970;45(239):13–23. Epub 1970/02/01. 5440182 10.1136/adc.45.239.13 PMC2020414

[11] Bonat S, Pathomvanich A, Keil MF, Field AE, Yanovski JA. Self-assessment of pubertal stage in overweight children. Pediatrics. 2002;110(4):743–7. . 12359788 10.1542/peds.110.4.743

[12] Desmangles JC, Lappe JM, Lipaczewski G, Haynatzki G. Accuracy of pubertal Tanner staging self-reporting. Journal of pediatric endocrinology & metabolism: JPEM. 2006;19(3):213–21. . 16607921 10.1515/jpem.2006.19.3.213

[13] Hergenroeder AC, Hill RB, Wong WW, Sangi-Haghpeykar H, Taylor W. Validity of self-assessment of pubertal maturation in African American and European American adolescents. The Journal of adolescent health: official publication of the Society for Adolescent Medicine. 1999;24(3):201–5. Epub 1999/04/09. . 10195803 10.1016/s1054-139x(98)00110-4

[14] Morris N, Udry JR. Validation of a self-administered instrument to assess stage of adolescent development. Journal of Youth and Adolescence. 1980;9(3):271–80. 10.1007/BF02088471 24318082

[15] Rasmussen AR, Wohlfahrt-Veje C, Tefre de Renzy-Martin K, Hagen CP, Tinggaard J, Mouritsen A, et al Validity of self-assessment of pubertal maturation. Pediatrics. 2015;135(1):86–93. 10.1542/peds.2014-0793 . 25535262

[16] Ji CY. Age at spermarche and comparison of growth and performance of pre- and post-spermarcheal Chinese boys. American journal of human biology: the official journal of the Human Biology Council. 2001;13(1):35–43. 10.1002/1520-6300(200101/02)13:1<35::AID-AJHB1005>3.0.CO;2-E . 11466965

[17] Nielsen CT, Skakkebaek NE, Darling JA, Hunter WM, Richardson DW, Jorgensen M, et al Longitudinal study of testosterone and luteinizing hormone (LH) in relation to spermarche, pubic hair, height and sitting height in normal boys. Acta endocrinologica Supplementum. 1986;279:98–106. Epub 1986/01/01. . 3465183 10.1530/acta.0.112s098

[18] Sivananthan T, Bathur F, Jimenez M, Conway A, Idan A, Handelsman D. Objective non-intrusive markers of sperm production and sexual activity. Asian J Androl. 2012;14(3):476–80. Epub 2012/04/24. 10.1038/aja.2012.2 . 22522506 PMC3720168

[19] Laron Z. Age at first ejaculation (spermarche)—the overlooked milestone in male development. Pediatric endocrinology reviews: PER. 2010;7(3):256–7. Epub 2010/06/09. . 20526238

[20] Sun Y, Tao F, Su PY. National estimates of pubertal milestones among urban and rural Chinese boys. Ann Hum Biol. 2012;39(6):461–7. Epub 2012/08/07. 10.3109/03014460.2012.712156 . 22862548

[21] Kettaneh A, Heude B, Lommez A, Borys JM, Ducimetiere P, Charles MA. Reliability of bioimpedance analysis compared with other adiposity measurements in children: the FLVS II Study. Diabetes & metabolism. 2005;31(6):534–41. 16357801 10.1016/s1262-3636(07)70228-8 PMC3305462

[22] Bremner WJ, Vitiello MV, Prinz PN. Loss of circadian rhythmicity in blood testosterone levels with aging in normal men. The Journal of clinical endocrinology and metabolism. 1983;56(6):1278–81. . 6841562 10.1210/jcem-56-6-1278

[23] Harwood DT, Handelsman DJ. Development and validation of a sensitive liquid chromatography-tandem mass spectrometry assay to simultaneously measure androgens and estrogens in serum without derivatization. Clinica Chimica Acta. 2009;409(1–2):78–84. 10.1016/j.cca.2009.09.003 . 19747904

[24] Singh GK, Balzer BW, Desai R, Jimenez M, Steinbeck KS, Handelsman DJ. Requirement for specific gravity and creatinine adjustments for urinary steroids and luteinizing hormone concentrations in adolescents. Ann Clin Biochem. 2015 10.1177/0004563215580385 . 25780247

[25] Singh GK, Jimenez M, Newman R, Handelsman DJ. Immunoreactive LH in long-term frozen human urine samples. Drug testing and analysis. 2013 Epub 2013/04/23. 10.1002/dta.1481 . 23606665

[26] Aksglaede L, Sorensen K, Petersen JH, Skakkebaek NE, Juul A. Recent decline in age at breast development: the Copenhagen Puberty Study. Pediatrics. 2009;123(5):e932–9. 10.1542/peds.2008-2491 . 19403485

[27] Kaitz M, Sabato R, Shalev I, Ebstein R, Mankuta D. Children's noncompliance during saliva collection predicts measures of salivary cortisol. Dev Psychobiol. 2012;54(2):113–23. 10.1002/dev.20580 . 21761405

[28] Mouritsen A, Soeborg T, Johannsen TH, Aksglaede L, Sorensen K, Hagen CP, et al Longitudinal changes in circulating testosterone levels determined by LC-MS/MS and by a commercially available radioimmunoassay in healthy girls and boys during the pubertal transition. Horm Res Paediatr. 2014;82(1):12–7. 10.1159/000358560 . 25033974

[29] Biro FM, Pinney SM, Huang B, Baker ER, Walt Chandler D, Dorn LD. Hormone changes in peripubertal girls. The Journal of clinical endocrinology and metabolism. 2014;99(10):3829–35. 10.1210/jc.2013-4528 25029416 PMC4184081

[30] Kushnir MM, Rockwood AL, Bergquist J, Varshavsky M, Roberts WL, Yue B, et al High-sensitivity tandem mass spectrometry assay for serum estrone and estradiol. Am J Clin Pathol. 2008;129(4):530–9. 10.1309/LC03BHQ5XJPJYEKG . 18343779

[31] Kushnir MM, Rockwood AL, Bergquist J. Liquid chromatography-tandem mass spectrometry applications in endocrinology. Mass spectrometry reviews. 2010;29(3):480–502. 10.1002/mas.20264 . 19708015

[32] Konforte D, Shea JL, Kyriakopoulou L, Colantonio D, Cohen AH, Shaw J, et al Complex biological pattern of fertility hormones in children and adolescents: a study of healthy children from the CALIPER cohort and establishment of pediatric reference intervals. Clin Chem. 2013;59(8):1215–27. 10.1373/clinchem.2013.204123 . 23637248

[33] Duke SA, Balzer BWR, Steinbeck KS. Testosterone and its Effects on Human Male Adolescent Mood and Behavior: A Systematic Review. Journal of Adolescent Health. 2014;55(3):315–22. 10.1016/j.jadohealth.2014.05.007 25151053

[34] Balzer BW, Duke SA, Hawke CI, Steinbeck KS. The effects of estradiol on mood and behavior in human female adolescents: a systematic review. Eur J Pediatr. 2015;174(3):289–98. 10.1007/s00431-014-2475-3 . 25567794

[35] Rosner W, Hankinson SE, Sluss PM, Vesper HW, Wierman ME. Challenges to the measurement of estradiol: an endocrine society position statement. The Journal of clinical endocrinology and metabolism. 2013;98(4):1376–87. 10.1210/jc.2012-3780 23463657 PMC3615207

[36] Marceau K, Ram N, Houts RM, Grimm KJ, Susman EJ. Individual differences in boys' and girls' timing and tempo of puberty: modeling development with nonlinear growth models. Dev Psychol. 2011;47(5):1389–409. Epub 2011/06/07. 10.1037/a0023838 . 21639623 PMC3928626

[37] Steinbeck K, Hazell P, Cumming RG, Skinner SR, Ivers R, Booy R, et al The study design and methodology for the ARCHER study—adolescent rural cohort study of hormones, health, education, environments and relationships. BMC Pediatr. 2012;12:143 Epub 2012/09/07. 10.1186/1471-2431-12-143 22950846 PMC3496596

